# Assessment of Virally Vectored Autoimmunity as a Biocontrol Strategy for Cane Toads

**DOI:** 10.1371/journal.pone.0014576

**Published:** 2011-01-25

**Authors:** Jackie A. Pallister, Damien C.T. Halliday, Anthony J. Robinson, Daryl Venables, Rhonda D. Voysey, Donna G. Boyle, Thayalini Shanmuganathan, Christopher M. Hardy, Nicole A. Siddon, Alex D. Hyatt

**Affiliations:** 1 Australian Animal Health Laboratories, CSIRO Livestock Industries, Geelong, Victoria, Australia; 2 Virology and Developmental Biology Group, CSIRO Entomology, Black Mountain Laboratories, Canberra, Australia; Cedars-Sinai Medical Center and University of California Los Angeles, United States of America

## Abstract

**Background:**

The cane toad, Bufo (Chaunus) marinus, is one of the most notorious vertebrate pests introduced into Australia over the last 200 years and, so far, efforts to identify a naturally occurring B. marinus-specific pathogen for use as a biological control agent have been unsuccessful. We explored an alternative approach that entailed genetically modifying a pathogen with broad host specificity so that it no longer caused disease, but carried a gene to disrupt the cane toad life cycle in a species specific manner.

**Methodology/Principal Findings:**

The adult beta globin gene was selected as the model gene for proof of concept of autoimmunity as a biocontrol method for cane toads. A previous report showed injection of bullfrog tadpoles with adult beta globin resulted in an alteration in the form of beta globin expressed in metamorphs as well as reduced survival. In B. marinus we established for the first time that the switch from tadpole to adult globin exists. The effect of injecting B. marinus tadpoles with purified recombinant adult globin protein was then assessed using behavioural (swim speed in tadpoles and jump length in metamorphs), developmental (time to metamorphosis, weight and length at various developmental stages, protein profile of adult globin) and genetic (adult globin mRNA levels) measures. However, we were unable to detect any differences between treated and control animals. Further, globin delivery using Bohle iridovirus, an Australian ranavirus isolate belonging to the Iridovirus family, did not reduce the survival of metamorphs or alter the form of beta globin expressed in metamorphs.

**Conclusions/Significance:**

While we were able to show for the first time that the switch from tadpole to adult globin does occur in B. marinus, we were not able to induce autoimmunity and disrupt metamorphosis. The short development time of B. marinus tadpoles may preclude this approach.

## Introduction

The spread of the cane toad, *Bufo (Chaunus) marinus*, into the Australian environment following the initial introduction at Gordonvale near Cairns, Queensland in 1935 has been spectacularly successful. Cane toads are now present throughout most of tropical northern Australia and their range is continuing to expand into world heritage areas. Predicted warming due to climate change could extend the range of the cane toad south into what are currently temperate regions [1]. The cane toad is a highly toxic and hardy introduced species and presents wide ranging ecological and social impacts within the Australian landscape. Native species such as quolls, goannas and native frogs are particularly susceptible to the cane toad toxin and many populations have been severely impacted by the arrival of cane toads [2], [3], [4].

Attempts to halt the spread cane toads have so far been unsuccessful mainly due to the extensive, remote and inaccessible areas inhabited by the toads. An infectious biological agent appears to be the only viable option for controlling cane toads at such continental scales but as yet no known naturally occurring microbes have been confirmed as *Bufo* specific. An alternative option is to explore whether an infectious agent can be genetically modified to carry a gene that will specifically disrupt the cane toad life cycle, requiring selection of cane toad specific target genes as well as an infectious agent for delivery.

The concept of using genetically modified infectious agents to deliver antigens to wildlife is not new. Recombinant vaccinia virus expressing rabies glycoprotein delivered in baits to wild foxes has proved to be a highly effective strategy to combat rabies [5]. Since then other vaccines developed against diseases of wildlife include a rabies virus based vector used to immunise wildlife against SARS [6]. Extension of this concept has seen recombinant viruses developed to control a host's biological processes. An example is recombinant viruses expressing zona pellucida antigen that successfully deliver immunocontraception to pest animal species in laboratory trials [7], [8].

Bohle Iridovirus (BIV) is a ranavirus in the family *Iridoviridae*. BIV was originally isolated from the Bohle River region in northern Queensland [9] and is the only documented isolation of a virus from amphibians in Australia. It is capable of infecting cane toad tadpoles [10] and is therefore a candidate for testing the viral delivery of genes in this species. Furthermore, in recent studies we have shown that BIV can carry and express foreign genes in vitro [11].

Selection of target genes for delivery to cane toads has focused on metamorphosis since it is a critical phase in the amphibian life cycle. Metamorphosis is characterised by rapid and extensive morphological changes [12], [13], accompanied by strong shifts in the expression of genes and proteins at the molecular level [14], [15]. Cane toad genes that are expressed in metamorphs but not in tadpoles therefore represent ideal targets to block and thus manipulate aspects of development. One documented example of the transition from a larval to an adult form in amphibians is haemoglobin [16] and we have used microarray analysis to establish that adult haemoglobin is significantly upregulated during cane toad metamorphosis [17]. Injecting tadpoles with adult globin interfered with expression of this protein in *Rana catesbeiana*, and induced changes in gene expression profiles of metamorphs [18]. Thus we hypothesise that it may be possible to alter metamorphosis by immunologically sensitising larval stages (tadpoles) to proteins expressed only in later post-metamorphic stages. Adult globin is not a cane toad specific gene; we used it here to determine whether autoimmunity might affect the protein profile of metamorphs. If successful, the concept would be extended to cane toad specific genes that were upregulated at metamorphosis.

This study outlines an investigation into the feasibility of an immunologically based biocontrol for cane toads. We demonstrate the presence of a clear larval to adult switch in haemoglobin mRNA and protein levels and hypothesise that this switch may be affected by early exposure, by either injection or viral delivery, to adult *B. marinus* haemoglobin. Our results indicate that the altered adult globin protein profile seen in *Rana catesbeiana* metamorphs after exposure of tadpoles to adult globin does not occur in *B.marinus*. The short larval stage in *B. marinus* compared with *R. catesbeiana* may preclude this approach to cane toad biocontrol.

## Materials and Methods

### Animals and husbandry

All animals used in these studies were sourced from a colony of *B. marinus* maintained at CSIRO according to the methods described in Hamilton et al. [19]. Briefly, when tadpoles were required, adults were injected subcutaneously with a 0.25 mg/mL solution of leuprorelin acetate to induce ovulation and stimulate amplexus. Eggs were hatched and tadpoles maintained in aged water without chlorine at a temperature of 23–27°C.

### Ethics statement

Authority for the use of animals was provided by CSIRO animal ethics committees in accordance with the Australian National Health and Medical Research Council's code of practice [20]. These permits were (i) CSIRO Sustainable Ecosystems Animal Ethics Committee, Approval No. 08-05, exposure of pre- and post-metamorphic cane toads to proteins, DNA and RNA and produced RNA/cDNA and (ii) CSIRO Australian Animal Health Laboratory Animal Ethics Committee, Approval number 1132, biological control of cane toads.

### Production and purification of recombinant globin and antisera

*B. marinus* adult and tadpole globins (GenBank Accession numbers EL342145 and EU877979, respectively) were amplified using the following full length primer sets: adult globin sense 5′- ATGGTCCATTTGACAGATCAC -3′, and antisense 5′- TTAGTGGTAACCCTTGCCAAG -3′ (444 bp), or tadpole globin sense 5′- ATGGTTCATTGGACCGCTGAAGA -3′, and antisense 5′- TTAGAAATAGCCATGGCTCAGG -3′ (444 bp). The fragments were cloned into the bacterial expression vector pDEST17 and expressed as His_6_-tagged proteins in *E. coli* BL21-AI cells (Invitrogen). Cultures were grown overnight (37°C) in LB supplemented with antibiotics, then diluted 100-fold and grown to an OD of 0.6 (600 nm). L-arabinose (Sigma) was added (0.2% final conc.) to induce protein production and incubation continued for 3–5 h. Bacteria were harvested by centrifugation, rinsed and resuspended in Tris-buffered saline (TBS: 50 mM Tris, 500 mM NaCl; pH 7.5), disrupted by freeze/thaw cycles and centrifuged at 10,000× *g* for 30 min. The pellet was solubilised in TBS containing 8 M Urea for 30 min and then centrifuged at 20,000× *g* for 30 min to remove insoluble materials. His_6_-tagged proteins were purified in the denatured state using Ni^2+^NTA agarose (Qiagen), washed via imidazole-containing steps (TBS+20, 30 or 40 mM imidazol) and eluted in TBS+500 mM imidazole. Size-based secondary purification was then achieved by continuous-elution electrophoresis (Model 491 Prep Cell, Bio-Rad). Globin proteins were dialysed against amphibian Ringers solution [4.89 g NaCl, 0.298 g KCl, 0.265 g CaCl_2_.2H_2_0, 0.197 g MgSO_4_.7H_2_0, 1.495 g NaHCO_3_, 0.127 g NaH_2_PO_4_.H_2_O and 1.982 g glucose per litre dH_2_O] overnight at 4°C and concentrations determined using the Bio-Rad Protein Assay. Proteins were separated by polyacrylamide gel electrophoresis (SDS-PAGE) (15% gels) using the Bio-Rad Protean II System and visualised with Coomassie brilliant blue. Purified recombinant globin proteins were used as immunogens for the generation of rabbit antiserum. Briefly, two rabbits per immunogen were injected 10 days apart with vaccine containing 50 µg of either adult (rAdglob) or tadpole (rTadglob) recombinant globin. Two weeks later a third dose was administered if a boost to the antibody response was required. Each dose was prepared in CSIRO triple adjuvant (60% v/v Montanide; 40%, v/v rHb [combined with Quil A, 3 mg/mL, and DEAE-dextran, 30 mg/mL]), in water.

### Developmental studies

The time course of adult and tadpole globin production in normal animals was determined. Animals were sampled at stages 27, 28, 34, 36, 40, 41, 42, 45, 46 (metamorph) and 1 month post metamorph, ≥3 animals per stage. Developmental stages of *B. marinus* were determined according to the method of Limbaugh and Volpe [21]. Levels of mRNA and protein were measured individually except for tadpoles at stages 27 and 28 where >10 animals were pooled due to the small size of tadpoles in the early stages of *B. marinus* development.

### Preparation of immunogen and inoculation trials

rAdglob in Ringer's solution was emulsified in Freund's complete adjuvant (FCA; Sigma) by Luer-locked double syringe mixing. Tadpoles were first anaesthetised by bathing for 2 min in 0.22% MS-222 (tricaine methanesulphonate, Argent Chemical Laboratories, Washington USA) and a 0.5 µg dose of rAdglob was delivered in 5 µL per stage 26 tadpole, intraperitoneally with a 30.5 gauge needle. Five animals were sampled and pooled at stages 26, 36, 40, 42, and 46. An additional 6 animals were sampled and assayed individually at stage 46.

### Recombinant virus construction

Recombinant BIV (rBIV) expressing the neomycin resistance gene and adult globin (rBIV/neo^r^/Adglob), and the control virus without adult globin (rBIV/neo^r^) were constructed according to Pallister et al. [11].

### Infection and sampling

To assess the effect of viral delivery of adult globin to *B. marinus* tadpoles, seven groups of tadpoles were infected at day 6 (stage 20) post hatching. Group A, uninfected cell culture supernatant; Groups B, C and D - 10^2^, 10^3^ and 10^4^ TCID_50_/mL of the negative control virus, rBIV/neo^r^ respectively; Groups E, F and G - 10^2^, 10^3^ and 10^4^ TCID_50_/mL of the test virus, rBIV/neo^r^/Adglob respectively. Each group of 116 tadpoles was infected for approximately 6 h in 2 L of water containing the appropriate concentration of virus, rinsed in clean water for 5 min then divided into 4 tubs each containing 29 tadpoles. These tubs were randomly dispersed around 3 different rooms to allow for statistical variation due to the position of the tub.

Sampling was carried out at 5 different stages, 20, 28, 33, 42 and 1–2 weeks post tail resorption, according to the schedule in Table 1. Tadpoles were sampled at each of the 5 stages for the detection of virus by real time PCR, and at all stages except stage 20, where tadpoles are very small, for the analysis of native adult globin profiles. However, tadpoles were only sampled up to and including stage 33 (just before the onset of native adult globin production) for the detection of viral adult globin. Five tadpoles per group were sampled at all stages except post tail resorption, where, for statistical purposes, 30 animals per group were sampled.

**Table 1:** Sampling schedule for tadpoles infected with recombinant BIV.

	Stage at sampling				
Sampled for	20	28	33	42	Post TR
Virus detection	√	√	√	√	√
Virus adult globin	√	√	√	-	-
Native globin	-	√	√	√	√

n = 5. Stage 20, infection; Stage 28, 6 days post infection; stage 33, just prior to the onset of adult globin production; stage 42, just after the onset of adult globin production; post TR, 1–2 weeks post tail resorption.

Tadpoles were euthanased by bathing for ≥5 minutes in 0.2% MS-222 (see Preparation of immunogen and inoculation trials). Tadpoles for real time PCR and for detection of viral adult globin were then frozen at −80°C until further processing. Tadpoles for blood sampling were euthanased as described then decapitated and blood was collected using a micropipette containing 0.2 M EDTA to prevent clotting. Red blood cells (RBCs) were pelleted at 13,000× *g* for 5 min, washed with PBS then lysed by resuspending in an equal volume of distilled water. Following centrifugation at 13,000× *g* for 5 min the supernatant containing globin was removed and stored at −80°C.

### Real time PCR to detect mRNA

Total RNA (5 µg) was extracted from tadpoles and toadlets using Trizol reagent (Invitrogen) according to the manufacturer's instructions and reverse transcribed using the cDNA First Strand Synthesis Kit (Invitrogen). Real time PCR was used to detect larval and adult globin mRNA at selected stages of development. Oligonucleotide sets were larval globin; sense 5′- GCTGAAGAGAAAGCCGC -3′, and antisense 5′- ATGGCGGTGACATTGGAC -3′ (151 bp), or adult globin; sense 5′- CAGATCACGAGCTCAAGAG -3′, and antisense 5′- ATGGCATCAGCAGAGCCA -3′ (151 bp). Results were standardised with cane toad actin (GenBank Accession no. EL595572) by amplifying a 117 bp fragment using sense 5′- ATGACACAGATAATGTTTGAGAC -3′ and antisense 5′- ATCACCAGAGTCCATCACAAT -3′ primers. Reactions consisted of QuantiTect SYBR Green RT-PCR Master Mix (Qiagen), 0.5 µM oligonucleotides and 200 ng of first strand cDNA template, and run on a RotorGene 2000 (Corbett). The thermal profile was as follows: 50°C for 2 min, 95°C for 2 min, 40 cycles of 95°C for 30 sec; 58°C for 30 sec; and 72°C for 30 sec. PCR target products were used as reaction standards at concentrations ranging from 1×10^2^ to 1×10^8^ molecules per µL. Analysis of real time PCR reactions and melting point dissociation curve reactions were performed using the programme Rotorgene version 6.0 (Corbett).

### Real time PCR to detect BIV DNA

For DNA extraction the animal was thawed, weighed and Prepman Ultra (Applied Biosystems) was added directly to the tube at the following rates: ≤0.20 g, 450–500 µL; 0.21–0.30 g, 500–750 µl; >0.31 g, 900 µL. This was followed by approximately 100 µL sterile 1.0 mm zirconia silicone beads (BioSpec Products). The tadpole was homogenised in a mini-beadbeater (BioSpec Products) for 30 sec, microfuged at 13,000× g for 30 sec then homogenised in a mini-beadbeater for a further 30 sec. To extract DNA the sample was heated to 100°C for 20 min, left to stand for 4 min at room temperature (RT), microfuged at 13,000× g for 6 min and the DNA (350–650 µl) transferred to a clean tube.

Each reaction in the TaqMan assay contained 12.5 µL of Platinum Quantitative PCR SuperMix-UDG (Invitrogen), 90 nM sense (5′- CTCATCGTTCTGGCCATCAA -3′), 90 nM antisense (5′- TCCCATCGAGCCGTTCA -3′) primers, 25 nM MGB TaqMan probe (5′- CACAACATTATCCGCATC- 3′), 5 µL of a 1/10 dilution of template DNA in dH_2_0, 0.05 µL of Rox dye, and water to a final volume of 25 µL. The reaction was run on the ABI 7500 Fast Real-Time PCR machine with a thermal profile was as follows: 2 min at 50°C, 10 min at 95°C, 45 cycles of 15 sec at 95°C then 1 min at 60°C. Results were automatically plotted by the Sequence Detection System Software version 1.3.1 (Applied Biosystems).

### Western blot analysis

Proteins for western blot were extracted either from Trizol samples according to the manufacturer's instructions, or from virally infected tadpoles homogenised in 10 mM Tris with 100 µL sterile 1.0 mm zirconia silicone beads (BioSpec Products). The tadpole was homogenised in a mini-beadbeater (BioSpec Products) for 30 sec, centrifuged at 13,000× g for 30 sec then homogenised in a mini-beadbeater for a further 30 sec. SDS was added to a final concentration of 5% and the homogenate heated to 100°C for 1 min. After 30 min at RT to allow SDS to penetrate the sample, the homogenate was centrifuged, the supernatant decanted, heated to 100°C for 1 min and stored at −80°C until used. To extract globin from RBCs, blood was processed as previously described (see *Infection and sampling*) and the protein content of each sample was determined by measuring absorbance at 280 nm using a NanoDrop Nd-1000 spectrophotometer (NanoDrop Technologies). Total protein and viral or native adult globin (200 ng/well) were analysed by polyacrylamide gel electrophoresis in the presence of SDS (SDS PAGE) on 12% Bis/Tris precast gradient gels (Invitrogen) and proteins were visualised by staining with silver nitrate, according to the procedure of Heukeshoven and Dernick [22]. Proteins were transferred to Hybond N (Amersham) and the transferred membrane blocked overnight at 4°C in blocking solution (PBS/0.05% v/v Tween 20 plus 5% w/v skim milk powder) then incubated for 1 h at RT in blocking solution containing a 1/1000 dilution of serum from rabbits inoculated with the *Bufo marinus* red blood cell lysate. After 3 washes the membrane was incubated for 1 h at RT in horse-radish peroxidase conjugated sheep anti-rabbit IgG (Millipore, MA, USA) diluted 1/1000. The membrane was washed and adult globin detected using enhanced chemiluminescence (ECL) according to the manufacturer's instructions (Amersham UK).

### ELISA to detect antibody to injected globin

Samples were collected from stage 46 metamorphs anaesthetised by bathing for 2 min in 0.22% MS-222 followed by decapitation. Samples were collected by aspiration and pelleting of RBCs prior to the collection of sera-like fluids. Adult blood samples were collected as per Zupanovic et al. [23]. Microtitre plates (Beckton-Dickonson) were coated with 50 µL/well of rAdglob (10 µg/mL) and positive control ovalbumin (4 µg/mL; Sigma) in Ringer's solution overnight at 4°C and washed (x3) in TBS with Tween-20 (0.05%; TBST). The plates were blocked for 2 h with 5% skim milk powder (Bio-Rad) in TBST at 37°C. Metamorph sera (treated and control) and adult toad α-ovalbumin control sera were serially diluted in 1% skim milk/TBS and applied to plates (50 µL/well). After incubation for 1 h at 37°C the plates were washed with TBST three times. Rabbit antisera against toad IgG (1∶1000; Zupanovic et al. [23]) was added in 1% skimmed milk/TBS, incubated for 1 h at 37°C and washed (x3) in TBST. Next, goat α-rabbit IgG (1∶2000; KPL) was added to each well, incubated for 1 h at 37°C and again washed (x3) in TBST. HRP-conjugated streptavidin (50 µL; KPL) was incubated for 30 min at RT, washed three times and developed with peroxidase substrate solution (50 µL of TBM; KPL) and the absorbance measured (405 nm).

### Fitness

Fitness was assessed using burst swim speed for tadpoles and maximum jump distance for metamorphs. At 11 days post injection (∼1 week prior to metamorphic climax/stage 46) average burst speed in tadpoles was measured according to the method described by Van Buskirk and McCollum [24] where specific speed is normalised to body length rather than measuring absolute speed. Temperature at the time of testing was constant at 23–24°C. Jump distances were calculated in stage 46 metamorphs approximately 2 days after removal from aquatic tanks according to Wilson and Franklin [25]. Jumps were recorded using a digital camera (Canon; 15 frames sec^−1^) and distances calculated with MouseZoom version 1.4 (Freeware 1998–2003) and Microsoft Windows Movie Maker version 5.1 (1998–2001). Absolute distances were determined from screen pixel units with conversion to mm via background graph paper. It was determined that optimal jumps were performed on plastic rather than paper.

## Results

### Larval and adult haemoglobin expression during normal cane toad metamorphosis

**Figure 1:**
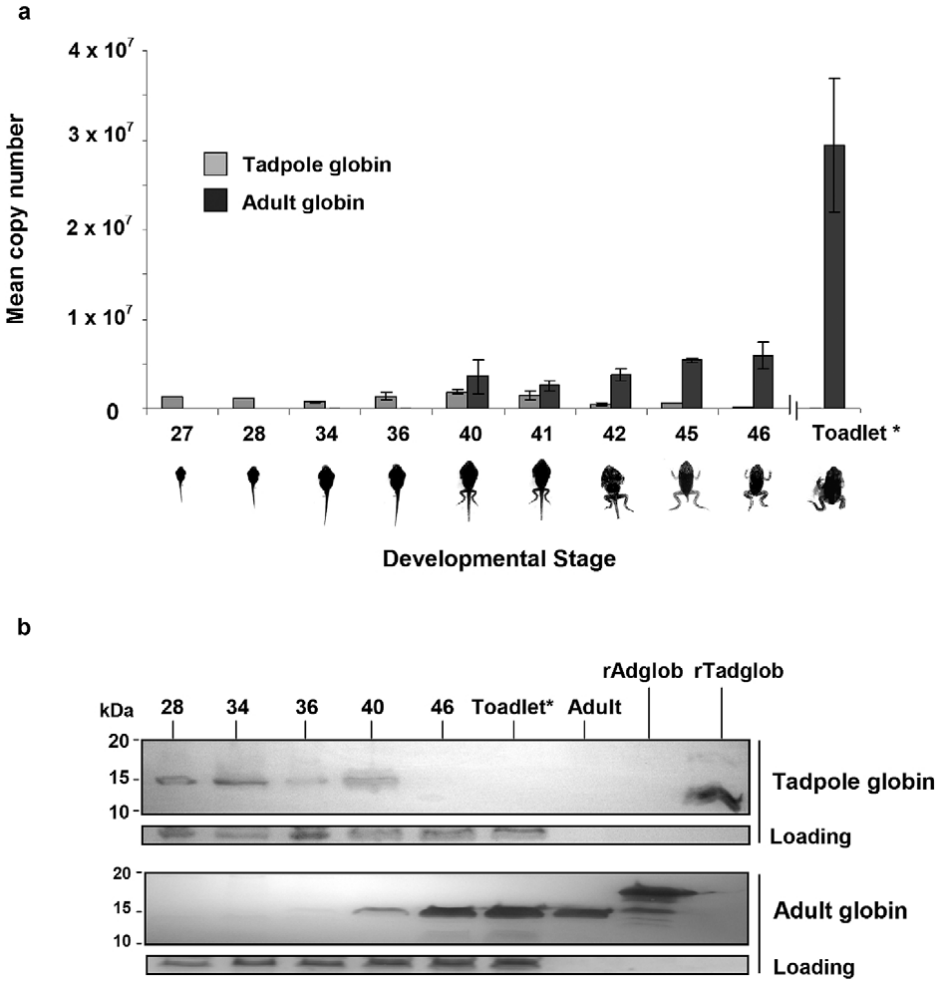
Tadpole to adult globin switch detected in normal cane toad development. a: mRNA data expressed as number of copies of adult or tadpole globin mRNA detected by real time PCR across various tadpole and metamorphic stages. Mean copy numbers were normalised using a toad actin housekeeping gene. Animals were staged according to Limbaugh and Volpe, 1957. Toadlet (*) development was approximately one month post-metamorphosis. b: Detection of globin proteins as determined by western blot analysis using specific antibodies to tadpole and adult globins. Coomassie staining indicates the loading level for each lane. Recombinant proteins for adult and tadpole globin (rAdglob and rTadglob, respectively), as well as native adult globin (Adult) were included as positive controls.

The time course for the appearance of adult globin mRNA and the disappearance of tadpole globin mRNA during normal cane toad metamorphosis were first assessed by real time PCR (Fig. 1a). Tadpole globin mRNA was detected at all early stages until the level declined rapidly at the metamorphic climax (Stages 42–46) and was undetectable one month after metamorphosis. Conversely, adult globin mRNA was first detectable at stage 40 and at all following stages until the experiment was terminated.

Analysis of total protein extracts at selected stages by Western blot showed that larval haemoglobin was expressed in stage 40 tadpoles but not in stage 46 toadlets (Fig. 1b). Adult globin protein was first detected in stage 36 tadpoles (faint signal) and continued to increase in abundance after metamorphosis. The positive control used for the experiments was recombinant adult globin that migrates as a larger protein than the native globin on PAGE gels due to the addition of a 6XHis-tag and plasmid linker sequence. The detection system was specific; anti-larval globin antibody detected larval globin, but not recombinant and native adult haemoglobins, and vice versa for the anti-adult globin antibody. Thus we confirmed that the globin switch seen at the mRNA level was also seen at the protein level.

### Effects of rAdglob injections on developing cane toad tadpoles

**Figure 2:**
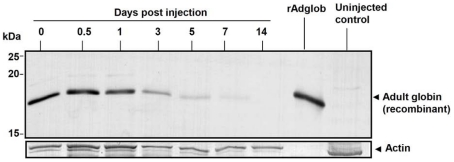
Time course detection of rHb within tadpoles after injection. Western blot using rabbit antibody to adult globin to detect persistence of rHb emulsion. n = 3 animals pooled per time point. Actin indicates loading per protein sample (mAb mouse anti-actin used at 1∶5000).

rAdglob within inoculated tadpoles was clearly detected by rabbit antibody to adult globin as an 18 kDa band for several days after injection (Fig. 2). rAdglob levels appeared reduced by half every 2–3 days, until 14 days post injection where no protein was detected.

**Figure 3:**
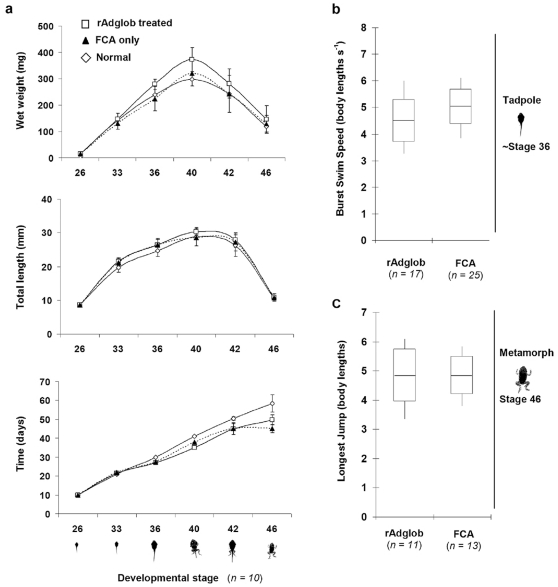
Physical measures of *B. marinus* development across metamorphosis unchanged by recombinant adult globin injection. a: Wet weights (mg), lengths (mm) and speed of development (days) for animals at six different developmental stages injected with rAdglob (□) or FCA control (▴, dotted line), as well as normal uninjected animals (◊) were recorded. n = 10 animals for each stage and error bars show standard deviation. b: Swimming performance of rAdglob treated and FCA control tadpoles at approximately stage 36 (or 10 days post injection) is shown. Burst swim speed represents the absolute swim speed normalised to body length, and the full data range (vertical line), standard deviation (box) and mean (horizontal line) are indicated. c: Jumping performance of rAdglob treated and FCA control amimals at stage 46 is shown. Longest jump distance was normalised to body lengths.

Gross morphology during metamorphosis determined by wet weight and length was similar for treated (rAdglob) and control groups (no injection, or Freund's adjuvant only) (Fig. 3a). Treatment with adult haemoglobin did not significantly delay metamorphosis. There was no significant difference in tadpole fitness between treatment and control groups just before metamorphic climax measured by swimming performance (burst swim speed). A p-value of 0.086 was determined in Microsoft Excel using the student t-test, 2-tailed distribution, 2-samples of unequal variance. Likewise there was no significant difference between treatments in the fitness of metamorphs as measured by maximum jump length (Fig. 3b). A p-value of 0.996 was determined using the same student t-test as for the swim speed.

**Figure 4:**
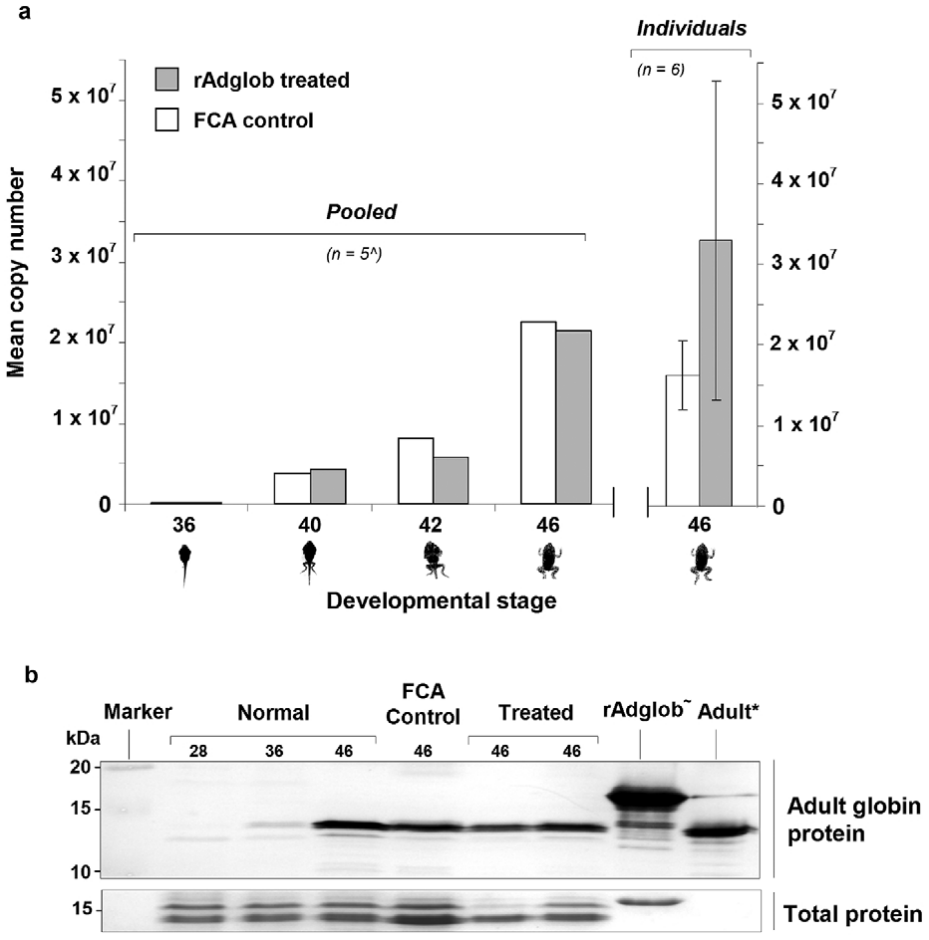
Adult globin mRNA and protein expression levels in treated and untreated *Bufo marinus*. a: The profile of adult globin mRNA expression across metamorphosis for treated rAdglob injected (grey shading) and control FCA only injected (white) animals was determined by real time-PCR analysis. Each stage point indicates the overall expression level for five pooled (∧) animals, with treated and control animals showing similar copy numbers. The last stage point represents an average of 6 individuals. Adult globin copy number was normalised to a toad actin housekeeping gene. b: Western blot analysis of whole animal extracts (upper panel) provides a general indication of the adult globin protein levels in normal developing animals (stages 28, 36 and 46), as well as FCA control and rAdglob treated animals. The lower panel shows the Coomassie brilliant blue staining for the same blot and provides an indication of total protein loading. rAdglob (∼) is an additional 3.3 kDa larger than native adult globin due to His_x6_ tag and linker. Adult (*) control was generated using purified globin from the RBCs of adult *B. marinus*. The blot is representative of rAdglob treatments (n = 10) and FCA controls (n = 9).

**Figure 5:**
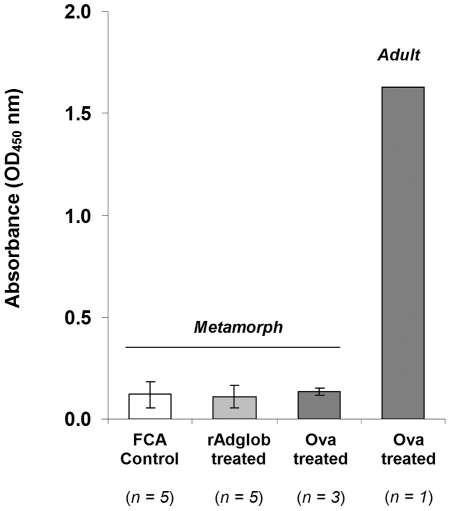
IgG antibody response to globin antigen not detected in metamorphs by ELISA. IgG levels as measured by ELISA in metamorphs (stage 46) injected with adult globin (rAdglob treated) compared with untreated metamorphs (FCA control). Positive controls show IgG levels in metamorphs and an adult injected with ovalbumin. Sera from rAdglob treated and FCA control animals used at 1∶80; ovalbumin treated metamorph sera used at 1∶320; ovalbumin treated adult sera used at 1∶1000.

Metamorphs from each of stages 36, 40, 42 and 46 were pooled and analysed for differences in adult globin mRNA between treated and untreated groups. The results indicated no significant differences in adult globin mRNA levels between treated and untreated groups at any of these 4 stages (Fig. 4a). A p-value of 0.914 was determined using the same student t-test as for the swim speed and jump length. As pooling animals from each stage could have masked effects in individual animals, 6 animals were taken from the treated and untreated groups at stage 46 and analysed individually. Again, no significant difference in globin mRNA levels was observed between individuals from treated and control groups. A p-value of 0.095 was determined using the student t-test outlined previously. As seen for the mRNA studies, we detected no change in the protein profiles of adult globin immunised metamorphs (n = 10) compared to control (n = 9) animals by Western blot (Fig. 4b). Similar results were recorded in preliminary trials conducted using native globin purified from the blood of adult toads rather than recombinant globin. These inoculations had no effect at the morphological or mRNA and protein levels (data not shown). We were unable to detect reactive antibodies (IgY) against recombinant globin protein in any of the animals by ELISA. However, we were also unable to detect antibody to a normally highly immunogenic test antigen (ovalbumin) in metamorphs, although reactive antibodies generated by immunising one adult with ovalbumin were readily detectable (Fig. 5).

### Viral delivery

The effect of recombinant virus infection on mRNA expression levels in tadpoles was assessed using real time PCR. Treated and control animal groups were sampled at stages 20, 28, 33, 42 and 1–2 weeks post tail resorption (Table 2). At stage 20, after infection and rinsing, no virus was detected in any animals indicating that there was no background level of BIV detectable by real time PCR and that any virus detected at later stages was the result of virus replication.

**Table 2:** Detection of viral DNA in treated and control animals.

	Animals positive by TaqMan PCR				
Group	Stage 20	Stage 28	Stage 33	Stage 42	Post TR
A: uninfected s/nate	0/5	0/5	0/5	0/5	0/5
B: rBIVneo^r^ 10^2^ dose	0/5	1/5	0/5	0/5	0/3
C: rBIVneo^r^ 10^3^ dose	0/5	2/5	0/5	0/5	0/3
D: rBIVneo^r^ 10^4^ dose	0/5	5/5	ND	ND	ND
E: rBIV/neo^r^/adglob 10^2^ dose	0/5	0/5	1/5	1/5	0/5
F: rBIV/neo^r^/adglob 10^3^ dose	0/5	2/5	2/5	4/5	0/3
G: rBIV/neo^r^/adglob 10^4^ dose	0/5	4/5	ND	ND	ND

Number of animals out of 5 sampled from each group that returned a positive result in TaqMan real time PCR for the presence of viral DNA. ND: not taken as tadpole numbers were reduced at the highest virus dose and preference was to maximise the number of animals for analysis of globin at 1–2 weeks post tail resorption (TR). C_t_ values of >35, corresponding to <10 copies of the BIV genome were considered indeterminate and recorded as a negative result.

BIV was detected in all of the infected groups, with more infected animals detected as the inoculum increased. The control virus (rBIV/neo^r^) was only detected at stage 28, 6 days post infection (pi), while the test virus (rBIV/neo^r^/Adglob) was detected at stages 28, 33 and 42 (day 6 pi to approximately day 26–30 pi).

### No effect of infection with rBIV/neo^r^/Adglob on adult globin protein profiles in metamorphs

**Figure 6:**
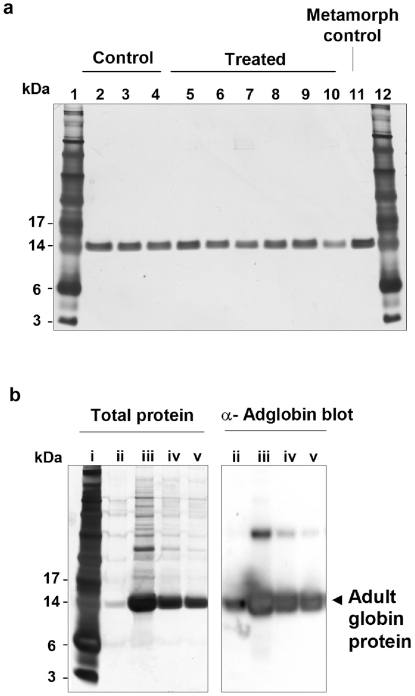
No effect on globin protein profile following infection of tadpoles with virus carrying rAdglob. 200 ng protein per well from lysed red blood cell samples taken from metamorphs then stained with silver stain. a: Lanes 1 and 12, See Blue Plus2 Pre Stained Standard (Invitrogen); Lanes 2–4, blood from 3 control animals bathed in 10^2^ TCID_50_/ml of rBIV/neo^r^; Lanes 5–10, blood from 6 test animals bathed in 10^2^ TCID_50_/ml of rBIV/neo^r^/adglo. Lane 11, positive control, globin from a 2 month old toadlet. b: Panel shows silver stain and Western blot antibody detection of adult globin; lane i See Blue Plus2 Pre Stained Standard (Invitrogen); lane ii, positive control, globin from a 2 month old toadlet; lanes iii, iv and v, 500, 1000 and 5000 ng of protein from fluid of untreated stage 42 tadpoles.

Analysis of adult globin profiles was carried out on RBCs from tadpoles taken at stage 42 and metamorphs at 1–2 weeks post tail resorption, by which time the switch has been made from tadpole to adult globin production. Blood from tadpoles given all three doses of the test virus (rBIV/neo^r^/Adglob that does express adult globin) and the control virus (rBIV/neo^r^ that does not express adult globin) were analysed by polyacrylamide gel electrophoresis (PAGE) and silver staining (Fig. 6a). The protein profiles of blood from animals infected with the rBIV/neo^r^ control (n = 51) and rBIV/neo^r^/Adglob (n = 89) viruses were all similar. A western blot using rabbit anti toad adult globin confirmed that the main 14 kDa band detected by silver stain was adult globin (Fig. 6b). We were thus unable to detect any change in the adult globin profile in RBCs taken from the animals that had been exposed to adult globin as tadpoles.

## Discussion

In this report we outline the steps we have undertaken to determine whether interference with cane toad tadpole development can be achieved using an immunological approach. Our proof of concept approach was largely influenced by previous observations that in bullfrog tadpoles immunised with purified adult globin, the adult globin protein profile was altered in surviving metamorphs [18]. Here we extended the concept to test whether a similar effect could be induced in *B. marinus* by injecting tadpoles with purified native and recombinant globin as well as viral delivery of this antigen.

We first established that the larval to adult globin switch reported in other amphibian species also occurred in *B. marinus.* It is well documented that a tadpole form of globin in anurans is replaced by adult globin during the course of metamorphosis [18], [26], [27] and here we demonstrate the existence of this switch for the first time in a *Bufo* species. We have previously demonstrated strong upregulation of adult globin genes during cane toad metamorphosis and that this was more pronounced than for any of the other genes induced at metamorphosis [17]. We have also previously shown that the recombinant BIV can be genetically modified to express adult globin *in vitro* [11] and confirmed here that this virus (rBIV/neo^r^/Adglob) is capable of infecting cane toad tadpoles. We therefore used rBIV/neo^r^/Adglob to assess the effect of viral delivery of an adult specific gene or protein to tadpoles on subsequent metamorphosis, and compared this to the effects of immunisation with purified protein and adjuvants.

A number of parameters were used to assess the effect of adult globin delivery to tadpoles. These included behavioural (average burst speed in tadpoles and maximum jump length in metamorphs), developmental (time to metamorphosis, weight and length at various developmental stages, protein profile of adult globin by PAGE) and genetic (mRNA levels for adult globin) measures. However, we were unable to detect any differences between treated and control animals following immunisation with purified globin or exposure to recombinant virus. This contrasts markedly with the effects of globin immunisation in *R. catesbeiana* reported previously by Maniatis et al. [18], who speculated that some form of immune response was instrumental in the altered adult globin profile observed in injected tadpoles.

Possible explanations for the differences observed between these studies is that tadpoles of *B. marinus* are inherently less immunocompetent than those of *R. catesbeiana* and/or they had less time than *R. catesbeiana* to mount an effective immune response to the globin antigen. Firstly, the cane toad has a very short larval stage of approximately 50–60+days, depending on tadpole density and temperature [28] whereas the larval stage in the bullfrog lasts at least 120 days and up to 2 years depending on environmental conditions [29]. The long larval stage in the bullfrog enabled an initial immunisation using FCA, followed by a boost 1 month later [18]. By contrast, cane toad tadpoles in our study were only large enough to be first injected at stage 26 (approximately day 9), barely 3 weeks before the onset of adult globin synthesis at stages 36–40 (day 30–43) and so a similar boost was not given. Nevertheless we considered that there should be sufficient time for a primary immune response to develop before adult globin appeared, provided cane toad tadpoles recognise the adult globin as a foreign protein. Secondly, due to the large difference between the cane toad and bullfrog developmental time frames, and as age at inoculation was not specified, the bullfrog tadpoles may have been inoculated later in development and been more immunocompetent than in our study. In support of this, studies in *Xenopus* have demonstrated that immune responses improve with age. The affinity of specific IgY antibodies against dinitrophenol (DNP) in *Xenopus* larvae is reported to be less than in adults, and in turn much lower than the affinity of mammalian anti-DNP IgG antibodies [30], [31]. The range of antibodies produced to DNP were also less heterogeneous in larval than in adult *Xenopus* [32].

Cell mediated immunity may also be impaired in tadpoles. In mammals the antiviral response relies on cytotoxic T lymphocytes and these are Major Histocompatibility Complex Class I (MHC class I) restricted. Larval *Xenopus* reportedly lack MHC classical class I expression [33] suggesting the antiviral response in larvae may be compromised. Studies of the adaptive immune response in *Xenopus* adults and larvae to frog virus 3 (FV-3), the type virus of the ranavirus genus, indicate this may be so. While adult *Xenopus* cleared an initial infection and showed an accelerated response to a second injection, tadpoles were much more susceptible, suffering a high mortality rate and a reduced ability to clear the infection compared with infected adults [34]. In spite of lacking MHC class I, tadpoles do have CD8 T cells [35] and so the role played by the lack of MHC class I in the poor antiviral response is unknown. While most of these studies have been carried out in *Xenopus*, limited studies indicate bullfrogs are capable of mounting a detectable antibody response to an antigen, but apparently not to influenza virus despite repeated inoculations and a substantial and anamnestic response to bacteriophage T7 [36]. Our own studies indicate that *B. marinus* tadpoles did not mount a detectable antibody response to a widely used and well characterised immunogen, ovalbumin, to which adult *B. marinus* did respond.

Precedents do exist for immune interference in development. Arif et al. [37] showed that when affinity purified antibody to a protein involved in insect metamorphosis was injected into late stage larvae, the development of the larvae into adult moths was defective. Other studies in rabbits [38] and mice [7] have shown that the immune response to an antigen can be enhanced by viral delivery.

However, all indications are that targeting autoimmune responses in larval amphibians may not be a useful strategy as the capacity of tadpoles to respond to immunological stimuli may be too weak to affect the chain of events at metamorphosis.

In conclusion, we have shown that the globin switch occurs in *B. marinus* and, while we have not been able to perturb this switch immunologically, it remains a viable target for other approaches such as RNA interference. Given the short larval phase in the *B. marinus* life cycle, antigens produced later than globin may be more effective immunogens and we are currently investigating this possibility. It is also possible that this approach would be more successful in an amphibian with a longer larval phase. Finally, we have designed, developed and delivered a recombinant viral system that will enable the development of future strategies to prevent the spread of the toad.
